# Prevalence estimation of *Pestivirus scrofae* (atypical porcine pestivirus) among Hungarian pig herds and the effects of different sample types on detection rates

**DOI:** 10.1186/s40813-024-00416-3

**Published:** 2025-02-14

**Authors:** Lilla Dénes, Mihály Albert, Barbara Igriczi, Gyula Balka

**Affiliations:** 1 Department of Pathology, University of Veterinary Medicine, István Str. 2., Budapest, 1078 Hungary; 2 CEVA-Phylaxia (Ceva Sante Animale), Szállás Str. 5., Budapest, 1107 Hungary

**Keywords:** Atypical porcine pestivirus, Genetic variability, Noninvasive sample types, Viral prevalence

## Abstract

**Background:**

Atypical porcine pestivirus (APPeV), also known as *Pestivirus scrofae*, is a member of the *Pestivirus* genus within the *Flaviviridae* family. Experimental infections have directly linked APPeV to congenital tremor (CT) type A-II in congenitally infected piglets born to challenged sows. Here, we report the assessment of the prevalence of APPeV in Hungarian pig herds and the influence of different sample types on detection rates.

**Results:**

Altogether, 2650 blood serum, 198 oral fluid and 163 processing fluid samples were obtained via a systemic approach from 26 Hungarian farms and one Slovakian farm. The samples originated from different age groups and were analyzed via reverse transcription–quantitative polymerase chain reaction (RT–qPCR). The estimated prevalence of APPeV was determined to be 66.67% in the sampled farms, indicating the widespread distribution of the virus within Hungary. Within the positive farms, APPeV genetic material was detected in the serum (21%), processing fluid (57%), and oral fluid (72%) samples. Notably, in some farms, the presence of APPeV was confirmed in only specific sample types, and five farms had APPeV in all three sample types. Age group analysis revealed that 10-week-old animals had the highest positivity rate in their blood serum (27%), whereas 20-week-old animals presented the highest rate in their oral fluid samples (59%). Processing fluid and oral fluid samples proved to be valuable for noninvasive diagnostic matrices, allowing for efficient population-level virus detection. We determined the partial NS2–3 coding region of 15 Hungarian strains and a Slovakian strain, and our phylogenetic analysis revealed that very similar strains can be found on different farms.

**Conclusion:**

In conclusion, our study provides insights into APPeV prevalence in Hungarian pig herds, emphasizing the importance of different sample types for accurate diagnostics. These findings contribute to our understanding of the virus's distribution across different age groups.

**Supplementary Information:**

The online version contains supplementary material available at 10.1186/s40813-024-00416-3.

## Background

Atypical porcine pestivirus (APPeV), also known as *Pestivirus scrofae*, is a member of the *Pestivirus* genus within the *Flaviviridae* family [1]. The virus was discovered in 2015 in the United States through next-generation sequencing analysis of samples belonging to animals coinfected with porcine reproductive and respiratory syndrome virus (PRRSV) [2]. Subsequent investigations revealed the presence of APPeV across diverse regions, including Europe [3–12], Canada [13], Asia [8, 14–16], and Brazil [17]. Recent findings extend its presence to wild boar samples in Germany, Serbia [18], Spain [19], Italy [20], and South Korea [21].

Experimental infection of pregnant sows with APPeV-containing blood or tissue suspensions results in clinical signs of congenital tremor (CT) type A-II in their offspring, which are often complicated with splay legs [5, 22]. In an outbreak of CT, Groof et al. [5] reported an overall 26% mortality among suckling piglets, with 60% of those deaths attributed to clinical CT and APPeV infection. Another study conducted in the southern region of Brazil reported 30% mortality until weaning among piglets that were affected by CT and tested positive for APPeV [23]. Schwarz et al. [9] reported a 10% decrease in reproductive performance during an APPeV outbreak on a commercial pig farm.

Several studies have pinpointed high APPeV genome loads in specific tissues, including whole blood, tracheobronchial and mesenteric lymph nodes, the spleen, nasal swabs [22], tonsils, the thymus [7], the *arcus palatoglossus*, *lymphonodus mandibularis,* nasal and Brunner’s glands [6]. In a recent study, a wide-ranging, systemic distribution of the virus’s RNA was described, including in endothelial cells, fibroblasts, and smooth muscle cells [24]. APPeV has been detected in the tunica albuginea [24], interstitial region, myoid cells, spindle-shaped cells surrounding convoluted tubules, Leydig cells, and walls of medium-sized (noncapillary) arteries [25] via RNA in situ hybridization. Previous studies have confirmed [5, 9, 17, 26] the presence of APPeV in the semen of sexually mature boars, suggesting that infected males shed the virus in their semen, similar to several other members of the *Flaviviridae* family, such as CSFV [27], BVDV [28] and Zika virus [29], which may play a role in virus transmission.

According to previous reports, APPeV shows relatively high genetic diversity between strains identified in different countries [7–10, 30–32]. Based on the analysis of the N^pro^ coding region, multiple distinct APPeV strains were identified within the same commercial boar stud farm [17]. Through the screening of semen and preputial samples, the authors suggested that the presence of distinct variants in the same farm can be explained by the sourcing of boars from different locations. Other studies, however, analyzing serum, fecal, and saliva samples, were only able to identify highly similar strains within each investigated farm based on the analysis of the NS2–3 segment, the entire coding region, or the NS5b segment [5, 9, 10].

The prevalence of APPeV has been partially assessed in various countries, albeit with varying sampling approaches. Beer et al. [3] conducted an investigation involving 379 tonsil and 63 serum samples from diverse types of facilities. Their findings revealed a 9% (33/367) APPeV-positive rate in tonsil samples collected from a rendering plant, APPeV positivity in all tonsil samples (12/12) collected from a slaughterhouse originating from an organic farm, and a 22% (14/63) APPeV-positive rate in sera collected from breeding and young fattening pigs in Schleswig–Holstein. In addition, the screening of 369 serum samples from various German farms revealed a 2.4% prevalence of APPeV [6]. A comprehensive analysis spanning approximately 30 years and involving 1080 serum samples in Switzerland revealed an approximate 13% prevalence of APPeV in pigs designated for slaughter, with a 1% prevalence observed in breeding pigs [11]. In Southwest China, a study encompassing 21 farms examined 39 serum samples from CT-affected piglets and 126 serum samples from healthy piglets for APPeV and found a 43.6% APPeV positivity rate among CT-affected piglets, whereas healthy piglets were found to be free from the virus [33]. In Spain, a retrospective analysis of 642 samples collected between 1997 and 2016 revealed that 13.9% of these samples tested positive for APPeV [7].

APPeV diagnostics are mostly based on molecular detection of the virus since there are no commercially available ELISA kits on the market. Development of a universal, broad-spectrum molecular assay is difficult considering the high genetic variability of the circulating strains [34]. Primers targeting the 5' untranslated region (UTR) of the *Pestivirus* genome are reactive toward both APPeV and distinct CSFV (classical swine fever, *Pestivirus suis*) genotypes, leading to limited specificity [11, 35]. Despite its wide applicability, the sensitivity of a TaqMan-based RT–qPCR test [6] for APPeV detection has been questioned because of the high variability of the strains [7]. The results of Muñoz-González et al. [7] revealed that the virus was not detectable in the blood serum of 42.3% of the examined pigs affected by CT, which may indicate that (i) the virus was not present in the blood serum at the time of sampling, (ii) another, unidentified virus caused the CT (e.g., LINDA virus/*Pestivirus L* [36]), or (iii) considering the high variability of APPeV strains, the RT‒qPCR test based on the TaqMan test used was not sufficiently sensitive.

To the authors’ knowledge, there is a lack of systematic studies investigating the estimated prevalence of the virus in commercial, large-scale pig farms. Therefore, our primary objective was to screen Hungarian herds, with a specific emphasis on the use of different diagnostic matrices and the assessment of within-herd infection dynamics of the virus on different farms.

## Materials and methods

### Samples

**Fig. 1:**
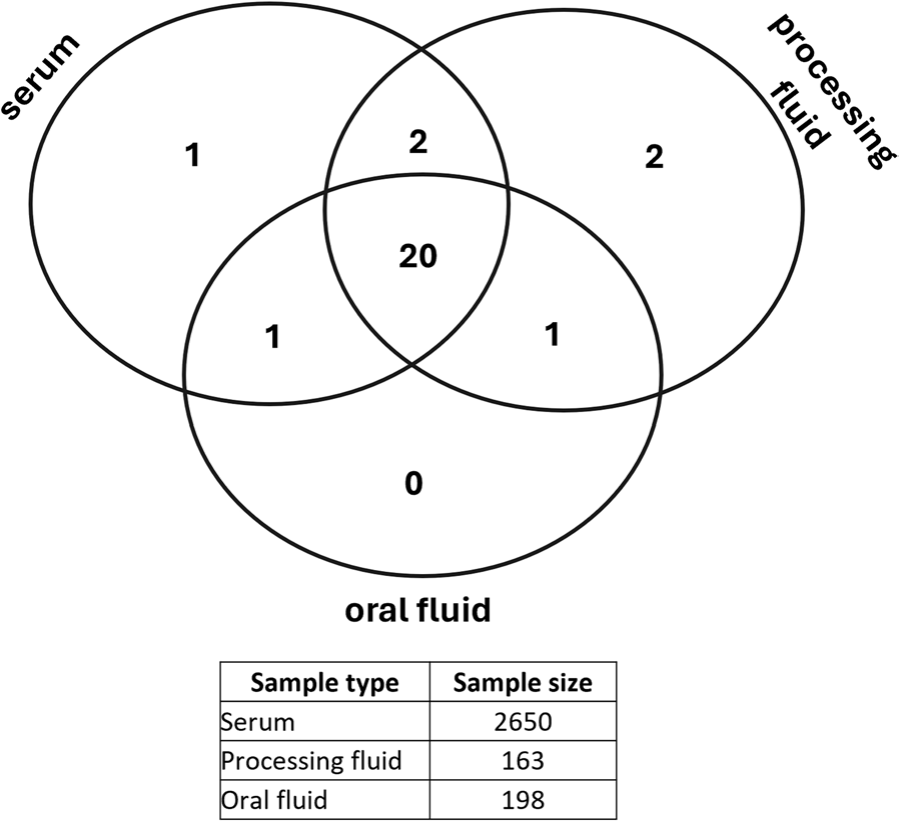
Number of farms shown concerning the collected sample types (serum and/or processing fluid and/or oral fluid) on a set diagram. Under the diagram, the number of samples/sample types is shown

We conducted extensive, systematic examinations on a total of 2650 blood serum samples originating from 26 Hungarians and one Slovakian farm in 2021 and 2022, which we also used for screening PCV3 [*Porcine circovirus 3*, [37]] and PPiV-1 [*Respirovirus suis*, [38]] (Fig. 1, S1). The study was conducted in compliance with the provisions of Directive 2010/63/EU, Hungarian Act XXVIII/1998 and the Hungarian Ministerial Decree No. 40/2013. (Ethical permission number: PE/EA/544-5/2018). These farms operated with 500–2000 sows at the time of our study. Specifically, we collected 100 samples (ranging from 60 to 160 samples) from 10 animals within each age group across 24 farms. The age groups subjected to analysis included 2-, 4-, 6-, 8-, 10-, 12-, 14-, and 18-week-old animals, as well as gilts and sows of two or four parity. These samples were organized into pools of five samples, each based on the respective age groups of the animals. For one farm, we lacked age group data for the 265 blood serum samples previously collected for PRRSV monitoring.

For 22 of these farms, we also conducted examinations on 163 processing fluid samples. Conversely, for two other farms, only processing fluid samples were analyzed. The processing fluid sample refers to the serous liquid obtained following the castration of male piglets aged 3–5 days, sourced from 10 to 15 different litters in our case. The collection process involved attaching a disposable plastic bag and a cloth diaper to a clean plastic bucket via a rubber band. The processed samples, including the collected tissue and fluid, were then placed on this setup. The cloth diaper, along with any remaining tissues at the end of the filtration process, was subsequently removed. Finally, the liquid collected in the plastic bag was transferred into plastic centrifuge tubes, each with a capacity of 15 ml, following a method described by López et al. [39].

We also collected a total of 198 oral fluid samples from 22 farms, usually 4 to 10 samples from each herd. These samples were obtained from 10-week-old animals in the battery/follow-up rearer phase and 20-week-old animals in the fattening farm stage. During the sample collection process, we suspended a specifically designed cotton rope above the group of animals for approximately 15–20 min, allowing each animal an opportunity to chew on it. We subsequently placed the ropes into a plastic bag, squeezed out the liquid content, and transferred it into a 15 ml sealed collection tube. These tubes were then transported to the laboratory for further testing [40]. All collected serum, processing fluid, and oral fluid samples were meticulously stored at − 80 °C until subsequent analysis.

Overall, we included 26 Hungarian and 1 Slovakian farm in our study to investigate the presence of APPeV (Fig. 1, S1).

### Viral RNA extraction and PCR

Viral RNA was extracted from the serum, processing fluid, and oral fluid samples via the Indispin/Cador Pathogen Mini Kit (Qiagen, Hilden, Germany) using the QIAcube (Qiagen) automatic nucleic acid extraction robot according to the manufacturer's instructions. The nucleic acids extracted from the samples were eluted in 60 μl of elution buffer (EB) (Qiagen) for each sample and then immediately subjected to RT‒qPCR analysis or stored at − 80 °C until further examination.

### RT‒qPCR

Reverse transcription–quantitative polymerase chain reaction (RT–qPCR) was used to analyze the serum, oral fluid, and processing fluid samples. A multiplex TaqMan assay [32], which employs fluorescently labelled probes designed for the NS3 and NS5b regions (Table 1), was applied.

**Table 1:** Primer and TaqMan probe sequences used for the detection and sequencing of the APPeV genome

Primer	Primer sequence	Target region	Purpose	Publication
NS3-F2 Q	GTGGTCATAGAYACYATGCAG	NS3	Diagnostics	[32]
NS3-R2 Q	TTCCTCTGGCCCTGTTCTTC	NS3	Diagnostics	[32]
NS3-P2 Q	FAM-TAGTGAATTTCTCVGCAAAGATGCC-BHQ1	NS3	Diagnostics	[32]
NS5B-F Q	TCGTCACTTAYAAGAAACCACG	NS5b	Diagnostics	[32]
NS5B-R Q	TTTACCCACTTGTACATTATTTTGGT	NS5b	Diagnostics	[32]
NS5B-P Q	FAM-ATACAGTACCCTGAGGCAGTCAC-BHQ1	NS5b	Diagnostics	[32]
APPV_4186-fw	GTGCGGCCTCCCAACTGTAG	NS2	Sequencing	[6]
APPV_4273-fw	TGGGGACCTCACCAGTGATG	NS2	Sequencing	[6]
APPV_5169-rev	ACGTCACCCTCTTTCCGCTC	NS3	Sequencing	[6]
APPV_5087-fw	GAAAGTGTCTGCCGCTTCATG	NS3	Sequencing	[6]
APPV_5703-rev	ACCATAYTCTTGGGCCTGSAG	NS3	Sequencing	[6]

RT–qPCR was carried out with a One Step RT-PCR Kit (Qiagen, Hilden, Germany) in a 25 μl reaction mixture comprising 7.5 μl of RNase-free water, 5 μl of 5 × QIAGEN OneStep RT‒PCR mixture, 1 μl of 10 mM dNTPs, 2.5 μM end concentration of each primer, 1.25 μM end concentration of each probe, 0.1 μl of RI (Ribolock, 40 U/µl, Fermentas, Waltham, USA), 1 μl of QIAGEN OneStep RT-PCR Enzyme Mix, 5 μl of template with the following temperature profile: reverse transcription at 50 °C for 40 min, inactivation of the reverse transcriptase enzyme and heat activation of Taq polymerase at 95 °C for 15 min, followed by 45 cycles of denaturation at 95 °C for 15 s, primer annealing and elongation at 58 °C for 30 s. The fluorescence data were collected during the final step of the cycles at an emission wavelength of 510 ± 5 nm, which corresponds to the FAM (6-carboxyfluorescein) dye, using the green channel on a Rotor Gene Q (Qiagen) device.

RT-PCR was applied with a One Step RT‒PCR Kit (Qiagen, Hilden, Germany) on samples positive for APPeV (Cq value ≤ 30), using sequencing primers targeting the NS2–3 region (Table 1). The process was carried out in a 50 μl reaction mixture comprising 28.5 μl of RNase-free water, 10 μl of 5 × QIAGEN OneStep RT‒PCR mixture, 2 μl of 10 mM dNTPs, 0.2 μM end concentration of each primer, 0.5 μl of RI (Ribolock, 40 U/µl, Fermentas, Waltham, USA), 2 μl of QIAGEN OneStep RT‒PCR Enzyme Mix, 5 μl of template with the following temperature profile: reverse transcription at 50 °C for 30 min, inactivation of the reverse transcriptase enzyme and heat activation of Taq polymerase at 95 °C for 15 min, followed by 40 cycles of denaturation at 95 °C for 30 s, primer annealing at 58 °C for 30 s and elongation at 72 °C for 30 s.

### Sequencing and phylogenetic analysis

For the PCR products obtained with the sequencing primers (Table 1), following agarose gel electrophoresis, amplicons of the appropriate lengths (NS2 ~ 800 bp, NS3 ~ 600 bp) were excised via a sterile scalpel. The gel fragments were subsequently purified via the Qiagen Gel Extraction Kit (Qiagen, Hilden, Germany) according to the manufacturer's instructions.

Bidirectional Sanger sequencing using two sets of overlapping primers (APPV_4186-fw/5169-rev or APPV_4273-fw/5169-rev and 5087-fw/5703-rev) targeting a 1635 nt long segment of the NS2–NS3 coding region (position of NS2 gene: 3394–4335 nt, NS3 gene: 4336–6395 nt [41]) was carried out by a commercial provider (Hungarian Natural History Museum, Budapest). The raw electropherograms were visualized via Chromas 2.6.6 software (Technelysium Pty Ltd., Brisbane, Australia), the reliability of the bidirectional sequencing results was compared, and a reference strain downloaded from GenBank (KY652092) was used; errors and discrepancies were corrected.

The obtained sequences were initially identified via BLASTn (NCBI) online software, and similar representative NS2–NS3 protein-coding sequences available in GenBank were collected. Sequence alignment was performed via the MAFFT 7 online software with the E-INS-i method [42]. Maximum likelihood (ML) analysis and phylogenetic tree reconstruction were conducted via MEGA X software [43], the appropriate model was selected with the MODELS setting [44], and ML bootstrap values were determined based on 1000 repetitions. The phylogenetic tree was visualized and edited via MEGA X software.

### Statistical analysis

The results obtained from the RT‒qPCR analysis of the serum, oral fluid, and processing fluid samples were recorded in tabular form. Statistical analysis was subsequently performed via GraphPad Prism 9 software (version 9.4.1.681, GraphPad Software, Boston, USA) to determine if there were significant differences among the various age groups. In the initial step, we examined whether a normal distribution was characteristic of our dataset for each set of data. If this assumption was met, a one-way ANOVA followed by Tukey’s post hoc test was conducted at the 95% confidence level. In cases where our data did not exhibit a normal distribution, a nonparametric Kruskal–Wallis test, followed by Dunn's post hoc test, was performed again at the 95% confidence level. Estimated true prevalence on a farm-level was determined using EpiTools (test sensivity: 0.9, test specificity: 0.99), Blaker’s confidence limits were calculated on a 95% confidence interval [45].

## Results

### Prevalence study

The presence of APPeV was assessed within blood serum, processing fluid, and oral fluid samples obtained from a combined total of 26 Hungarian and 1 Slovakian swine farm. Among the 26 farms included in the Hungarian prevalence survey, 18 farms were determined to be infected by RT–qPCR, leading to a calculated prevalence rate of 66.67%. The estimated true prevalence of the virus on farm-level was 13.9% (95% CI: 10.9%, 17.5%). Additionally, we identified the virus in samples originating from the Slovakian farm.

**Fig. 2:**
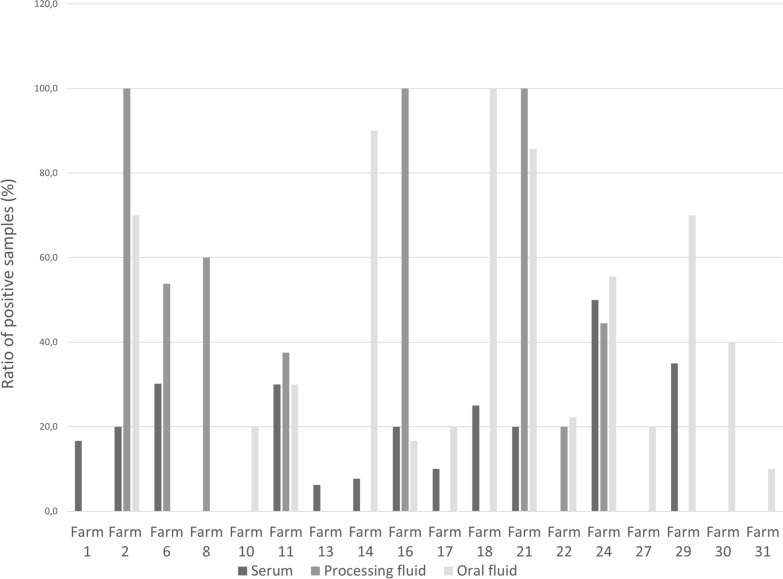
Proportion of positive serum, oral fluid and processing fluid samples in APPeV-positive farms

For the Hungarian farms that tested positive for APPeV, the genetic material of the virus was identified, on average, in 21% of the serum samples, 57% of the processing fluid samples, and 72% of the oral fluid samples. Specifically, the viral detection rate for the serum samples ranged from 6.3% to 50%, that for the processing fluid samples ranged from 20 to 100%, and that for the oral fluid samples ranged from 10 to 100% (Fig. 2). Notably, on four farms, the APPeV genome was detected only in oral fluid samples (Farms 10, 27, 30, 31), another four viruses were detected in serum and oral fluid samples (Farms 14, 17, 18, 29), and on one farm, the virus was detected in processing fluid and oral fluid samples only (Farm 22). In contrast, five farms demonstrated the presence of the virus across all three types of samples (Farms 2, 11, 16, 21, and 24). Additionally, two farms provided only processing fluid samples (Farms 7 and 8), whereas another farm sent us serum samples only (Farm 13); among these, two farms (Farms 8 and 13) were confirmed to be infected. Within infected farms where more than 5 processing fluid samples were collected and tested, the virus was consistently detected in all instances in this sample type (Fig. 2).

**Fig. 3:**
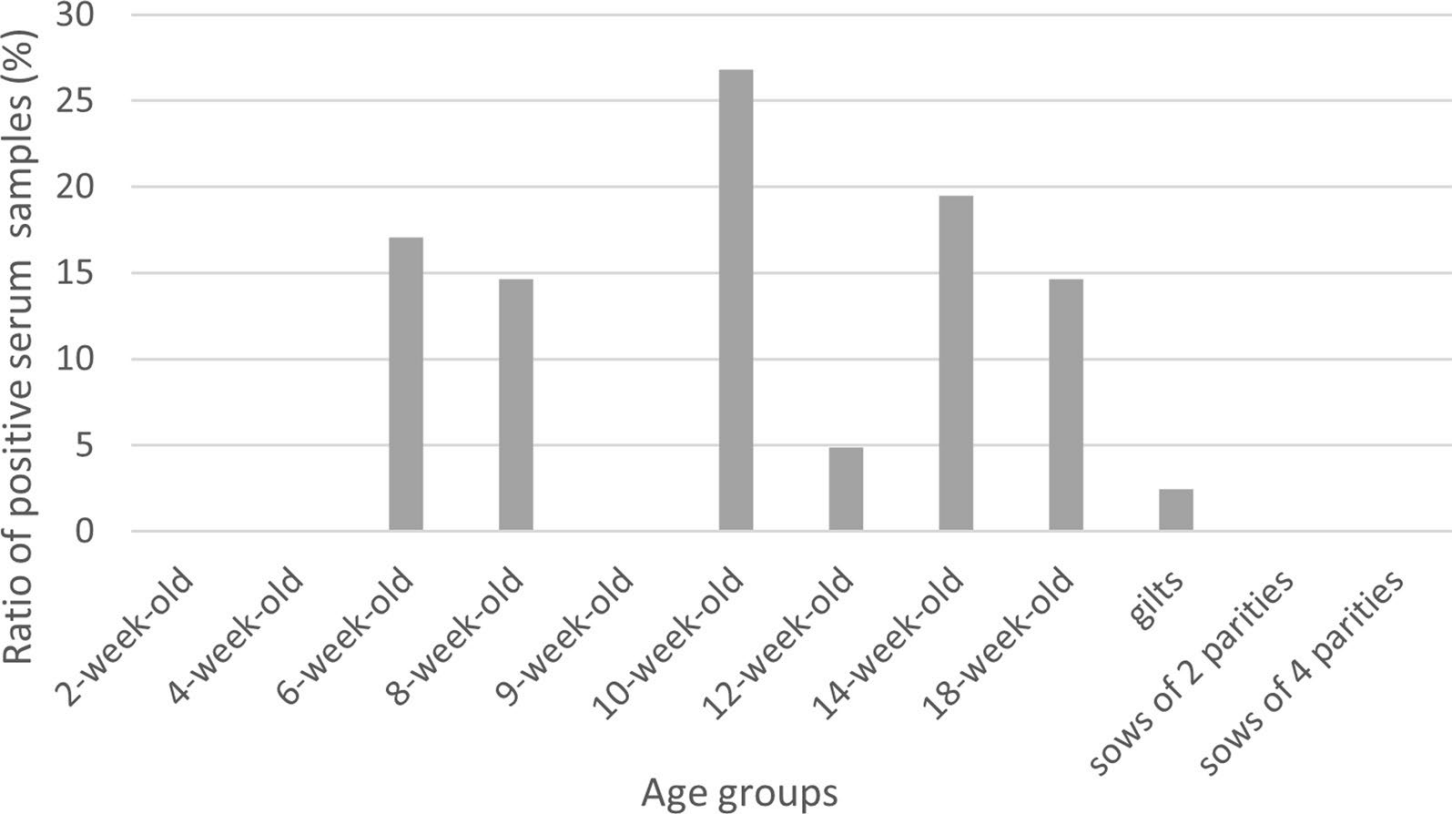
Percentage distribution of blood serum pools that tested positive for atypical porcine pestivirus (APPeV) among the examined age groups

For the farms that tested positive for APPeV, we conducted an analysis of infection dynamics in the different age groups (Fig. 3). Approximately 27% of positive serum samples originated from animals at 10 weeks of age, whereas approximately 17% positivity was observed in animals aged 14 and 18 weeks. Approximately 15% positivity was detected in animals aged 6 and 8 weeks, and approximately 4% positivity was detected in the serum pools of 12-week-old pigs. In samples collected from sows, the virus was identified in only approximately 6% of cases, specifically on two farms. Samples taken from sows that had multiple litters and animals younger than 6 weeks consistently tested negative.

**Fig. 4:**
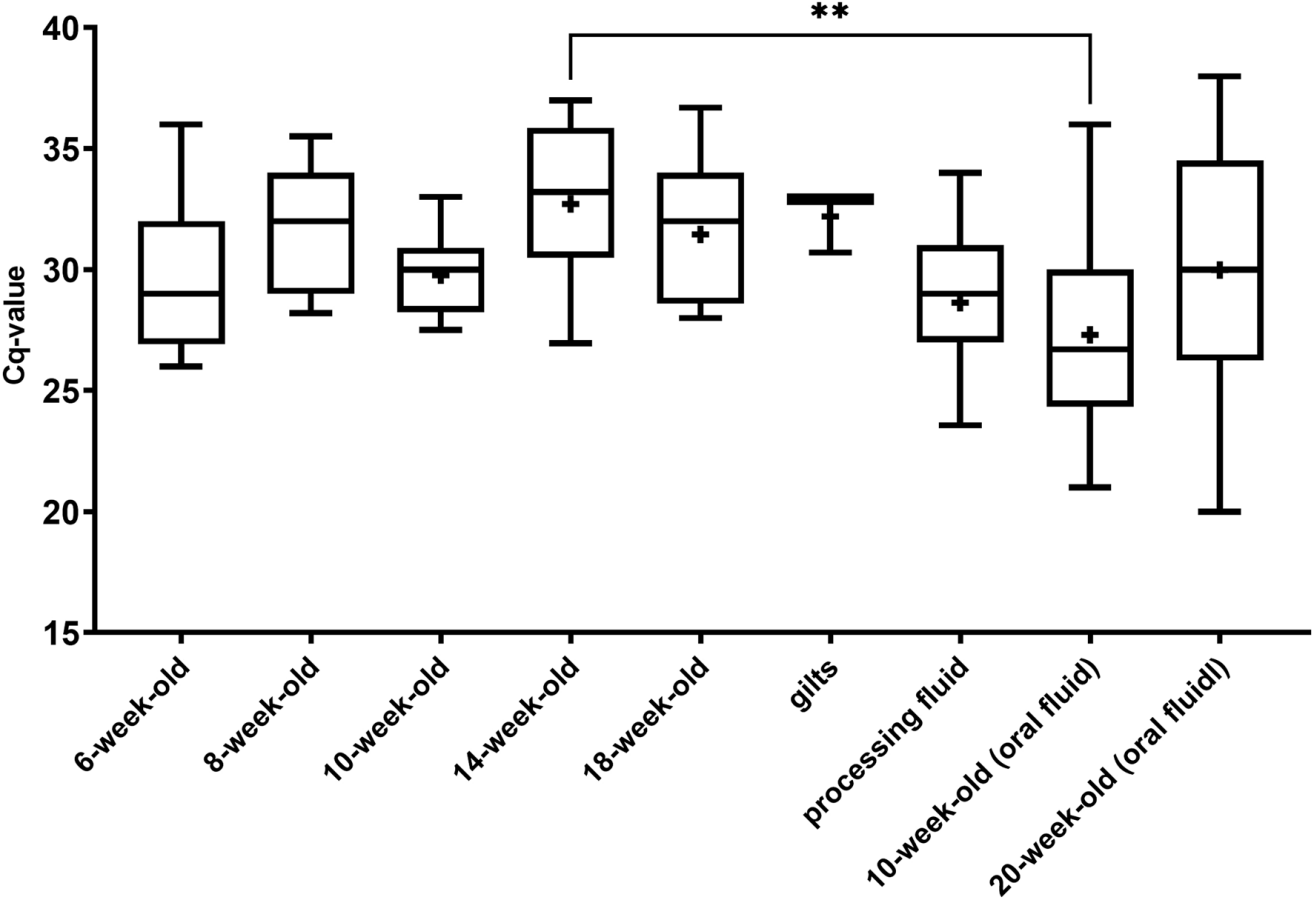
Boxplots comparing Cq values of positive serum pools, processing fluid, and oral fluid samples from different age groups. The whiskers indicate the minimum and maximum values, and the “+” signs indicate the sample means. The horizontal lines in the box represent the upper quartile, median, and lower quartile. Statistical comparison of Cq values was performed via one-way ANOVA. The asterisks above the boxes indicate statistically significant differences. (**: *p* < 0.01)

In terms of age groups, the Cq values (quantification cycles) of the samples were normally distributed, and the differences between the groups were thus analyzed via one-way ANOVA. We observed significant differences between the values within the groups; however, when comparing them, we found a significant difference (*p* < 0.0072) in the Cq values only between the serum samples taken from the 14-week-old animals and the oral fluid samples from the 10-week-old animals (Fig. 4).

**Fig. 5:**
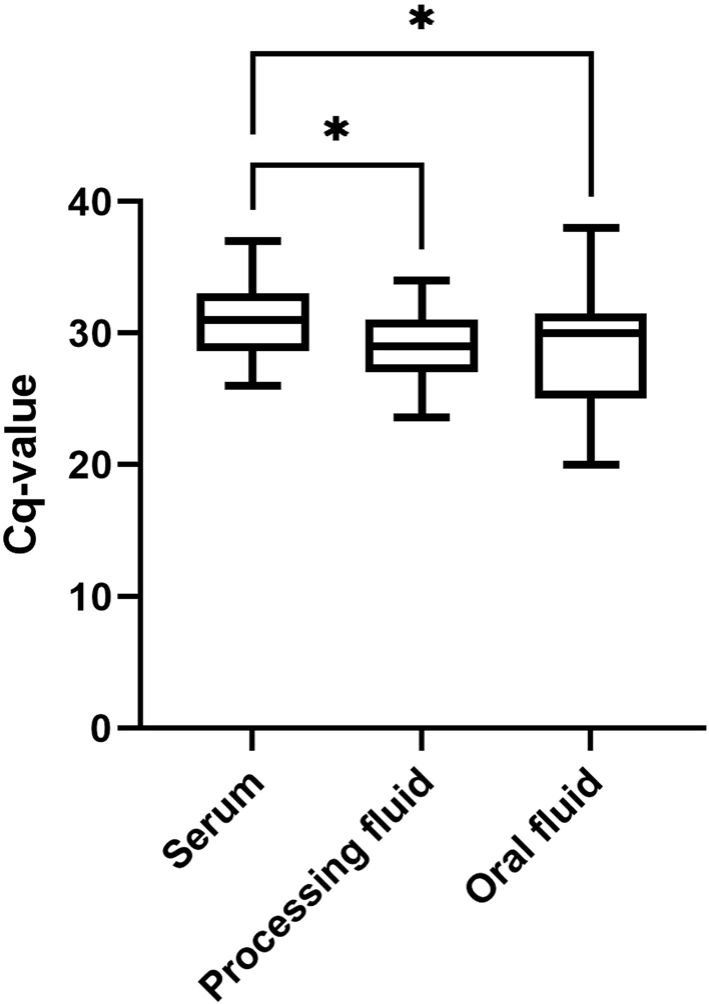
Comparison of the Cq values of different APPeV-positive samples by sample type, represented in a boxplot. The whiskers indicate the minimum and maximum values. The horizontal lines in the box represent the upper quartile, median, and lower quartile. Statistical comparison of Cq values was performed via one-way ANOVA. The asterisks above the boxes indicate statistically significant differences. (*: *p* < 0.05)

The Cq values of the different sample types were also compared via one-way ANOVA. We detected a significant difference between the serum-processing fluid samples (*p* < 0.0287) and the serum-oral fluid samples (*p* < 0.0137), based on which we established a greater Cq value in the serum samples (Fig. 5).

### Sequence analysis

The partial sequences of the NS2–3 protein-coding regions were determined from serum samples and added to a set of sequences obtained in our previous study [10]. A total of 15 farms where animals with CT clinical signs were examined (accession numbers: MH049523-33, OQ190176-83). These farms are indicated in Fig. 7.

**Fig. 6:**
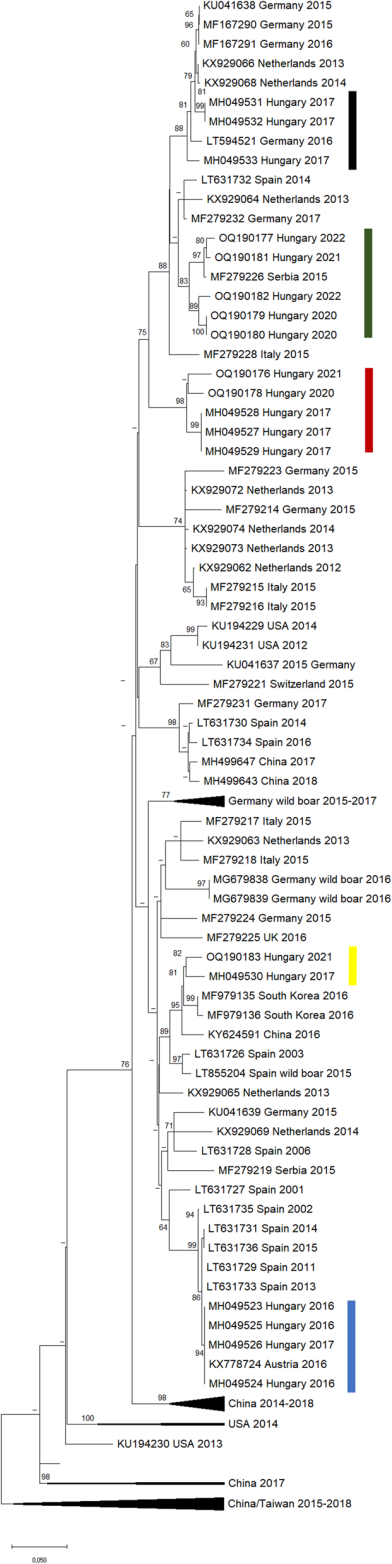
Phylogenetic tree based on the comparison of reference and our NS2–3 polypeptide-coding sequences of APPeV downloaded from GenBank. The comparison was conducted using the Maximum Likelihood method, and the resulting bootstrap values (≥ 70) are displayed above the branches as percentages. The scale indicates 0.01 expected changes at specific locations and branches. The coding of strains appearing on the phylogenetic tree is as follows: GenBank accession number_collection country_collection date. The vertical-colored bars correspond to the origin of Hungarian samples, as indicated in Fig. 7, using the same color scheme

Based on phylogenetic analysis, which included our recent and previous Hungarian sequences and a set of different APPeV strains, the virus shows high variability across countries (Fig. 6). In some cases, strains from a specific country form a distinct monophyletic group (as seen in Chinese sequences). The viral strains identified in Spain formed relatively small monophyletic groups, one of which presented relatively high similarity to a sequence identified at an Austrian pig farm (99.6%), and in Hungary (99.7%) in 2016 and 2017. The only known APPeV strain from a wild boar in Spain (Catalonia) exhibited high similarity to a sequence isolated from a Spanish pig farm 12 years later. Most strains collected from wild boars in Germany, regardless of collection time (2015–2017), form separate phylogenetic groups. However, three sequences from 2015 and 2016 presented greater similarity to certain strains identified in Italy, the Netherlands, and the United Kingdom (also from 2015 and 2016), as did a sample from a German pig farm identified in 2015.

**Fig. 7:**
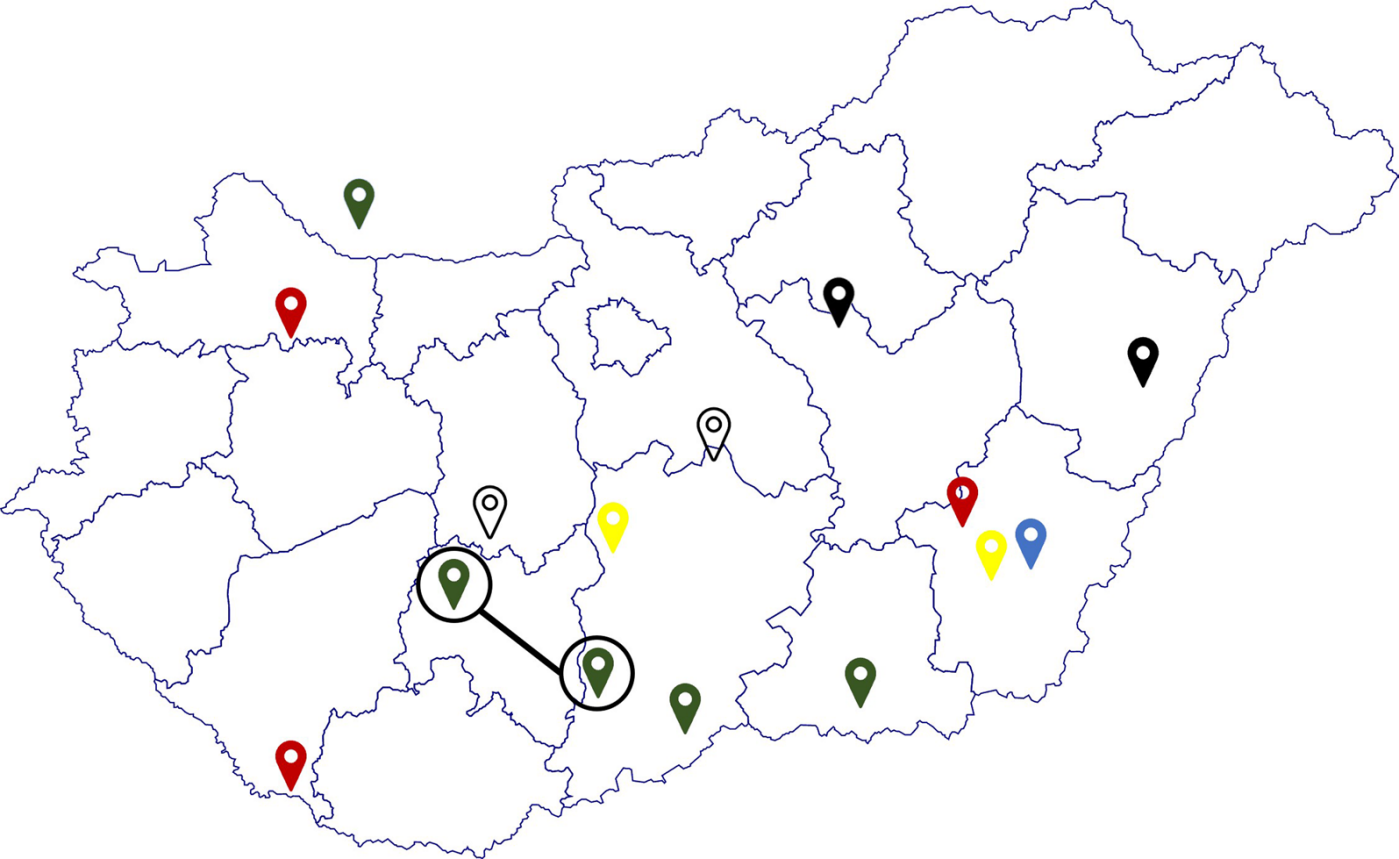
Color-coded representation of farms based on virus variants. Farms labelled with the same color indicate similar virus variants identified in those locations. Farms encircled and connected with a line denote the identification of the same virus variant in both locations

Strains identified in Hungary do not form separate groups based on individual farms (Figs. 6 and 7). Samples from two Hungarian farms located 130 km apart form a phylogenetic group with each other and with sequences from Germany and the Netherlands (black). A sample from Slovakia and one from southern China formed a smaller group with a strain identified in Serbia in 2015 (green). Three other strains from southern Hungary, originating from two farms approximately 100 km apart, belong to a broader phylogenetic group, where the sequences of the viruses identified in CT-affected piglets from two different farms are identical (green, black circle). An APPeV strain identified in 2017 from the organs of a CT-affected piglet forms a monophyletic group with a strain identified in two pig farms located 300 km away from each other 3–4 years later (red markers). Interestingly, two sequences identified in separate pig farms in Hungary also showed significant similarity (98.2%), as did a sample described in the Republic of Korea (yellow).

The greatest genetic distance was observed among Chinese strains (24.3%), which is notably greater than that reported in other countries (Germany, 13.9%; Italy, 11.3%; the Netherlands, 11.0%; Spain, 10.8%; USA, 13.9%). Within commercial Hungarian pig farms, genetic distances range from 1.1% to 11.2%, with an average genetic distance of 7.8%.

## Discussion

In this study, we aimed to (i) estimate the prevalence of APPeV infection in Hungarian large-scale industrial pig herds, (ii) investigate the within-herd infection dynamics of the virus, (iii) analyze the origin and phylogenetic relationships of the strains in the country and (iv) compare the utility of different sample types for the diagnosis of the virus.

In our investigation, we identified the presence of the virus in 18 out of 26 pig farms sampled; therefore, our prevalence survey of clinically healthy herds indicated a prevalence rate of 66.67%, suggesting a wide distribution of the virus within our country. In a retrospective Spanish study, APPeV was detected in pig serum samples dating back to 1997 [7], which remains the earliest documented instance of virus identification. Given the well-established history of CT in our country and globally, it can be speculated that the virus has indeed been present in pig populations for decades, manifesting symptoms sporadically. In contrast to our findings, the Spanish study reported 13.9% positivity in 642 serum samples spanning 1997–2016, with 57.7% of samples from CT-affected animals testing PCR positive for APPeV. In our laboratory, every CT-affected piglet we tested thus far was positive for the presence of the APPeV genome [10]. Although the correlation between APPeV and CT was evident, the present study also identified asymptomatic individuals infected with APPeV [7], similar to our previous observations [46]. Unfortunately, details regarding the number of farms participating in serum sample collection during the survey were not provided in this study. Among the 510 blood serum pools tested in our study, approximately 21% were positive for APPeV, which is 7% greater than that reported in Spain [7]. APPeV was detected on the majority of the investigated, still operating farms, where CT was previously observed. In the case of three farms, CT was observed in the past, but at the time of our study, we could not detect the virus via qRT-PCR. Presumably this deviation may be that we were only able to examine a smaller number of samples (12 serum pools, 5 processing fluid samples, and 10 oral fluid samples) at one of the mentioned facilities, and in two others, only oral fluid samples were examined. However, it cannot be ruled out that other, unknown factors were responsible for the symptoms. Notably, the sampling and viral detection methods used in our study had limitations. Most of the clinical samples were sent to our laboratory by local veterinarians, some of whom did not provide details on the size of the farms. Despite the large sample size, our investigation did not cover the entire country. Additionally, we did not use an internal control gene as part of the qRT-PCR system designed for APPeV detection, so there may be instances where we obtained false negative results.

In a considerable number of the farms assessed, the virus was detected in two of the following three sample types: blood serum, oral fluid, or processing fluid. There were only five farms, where APPeV was detected in all three sample types. In instances where the virus could be identified in serum but not in processing fluid, the number of processing fluid samples collected was five or fewer. Based on these findings, five or more processing fluid samples should be collected per farm for APPeV diagnostic purposes. This sampling procedure appears particularly effective for determining the APPeV infection status of a given pig farm, similar to PRRSV [46, 47]. This could be due to that the virus replicates in specific cells of the testicles in newborn piglets [46], making it suitable for identification not only in viremic animals.

Processing fluid is primarily used for PRRSV screening in newborn animals, as its presence in this age group indicates ongoing infection and virus circulation within the breeding herd [47]. Notably, although processing fluid samples from newborn animals confirmed the infection, no cases of congenital tremor were observed in the positive farms at the time of sampling. This discrepancy suggests that asymptomatic animals might have been infected later during their intrauterine development, during a period when nervous system infection did not result in CT symptoms. This interpretation is reinforced by our findings, where the virus was detected in the brain and thymus of a clinically healthy newborn piglet [46].

According to our results, the samples with the highest positivity rates were serum samples from 10-week-old animals (positivity rate of 27%) and oral fluid samples from 20-week-old pigs (positivity rate of 59%). In the context of the general screening of APPeV presence at a given farm, the examination of processing fluid samples and oral fluid samples can be recommended, as these sample types offer the advantages of noninvasive collection and screening of a larger population [48].

From serum samples obtained from 15 farms, we determined 22 partial NS2–3 protein-coding sequences, which were compared to each other and relevant APPeV sequences available in GenBank. The NS2–3 coding region is relatively conserved among *Pestiviruses* [6], therefore it is ideal for comparing genetically distant strains. Also, it is the most represented gene segment in the GenBank. In the case of the Hungarian strains, we found that very similar sequences originated from geographically distinct farms with no known epidemiological connections between them. Even complete sequence identity was found in the case of two sequences obtained from different farms. In cases where multiple samples were sequenced from a given farm, the 1635 nt long sequences were always identical. Unfortunately, we could not trace reliable information regarding live animal trade or other epidemiological connections among the farms included in our study, but the possibility that the farms had the same origin of gilt replacement or semen as potential sources of infection cannot be excluded.

The role of infected semen in the spread of APPeV has not yet been directly investigated [5, 9, 17, 26], but it is highly plausible that it also plays a role in virus transmission, as has already been reported for other pestiviruses [27, 28].

In a phylogenetic study conducted during a retrospective analysis of serum samples collected from pigs in Spain, the majority of the isolates presented significant genetic diversity and can be classified into various phylogenetic groups, along with different European and Chinese strains [7], similar to our findings regarding strains obtained from Hungary and Slovakia. Therefore, based on current data, APPeV strains cannot be classified according to their geographic origin. The greatest genetic distance was observed among the Chinese strains (24.3% difference), which was more than twice that reported in other countries. The high diversity of APPeVs could be explained by homologous recombination, as suggested by Guo et al., [49] similar to the closely related pestivirus BVDV (*Pestiviruses A* and *B*) [50].

The only known APPeV strain originating from a wild boar in Spain (Catalonia) shows a high degree of similarity with a sample isolated from a Spanish pig farm 12 years later that could indicate an epidemiological connection between wild boars and extensively kept Spanish Iberico pigs [51]. Several APPeV strains identified in wild boars in Germany were highly similar to strains described in domestic animals from Germany, Italy, the Netherlands, and Spain. A previous analysis of the mitogenome of wild boars in Europe revealed that the geographic distribution of the clades shows a clear phylogeographic pattern. They identified a contact zone of the three usual clades of Europe in Poland, two of which were also observed in Spain, Germany, Italy and the Netherlands; therefore, intensive gene flow took place in the east–west direction [52]. The genetic distribution of CSFV across Europe shows the same pattern [53]. Based on these observations, it is likely that wild boars play a role in the epidemiology of APPeV as virus reservoirs; hence, our future research will focus on extensive analyses of wild boar samples.

## Conclusion

This study elucidates the prevalence of APPeV within Hungarian pig populations, emphasizing the importance of employing diverse sample types to ensure precise diagnostic assessments. The results enhance our understanding of the virus distribution across different age groups and offer valuable perspectives for monitoring and managing its effects on pig herds. Our findings indicate that most strains lack geographic exclusivity, likely due to the trade of infected, asymptomatic animals.

## Supplementary Information

Supplementary Material 1: Table 1. Summary of the examined Hungarian farms, sample types and sample sizes. The number and proportion of APPeV-positive samples are also included in the table.

## Data Availability

Sequence data gathered during the study can be accessed under GenBank accession numbers: MH049523-33, OQ190176-83. Data regarding the name and exact location of the farms involved in the study are confidential due to business secret of the owners.
