# Supplementary material for: Prevalence estimation of Pestivirus scrofae (atypical porcine pestivirus) among Hungarian pig herds and the effects of different sample types on detection rates
Source: Porcine Health Manag. 2025 Feb 14;11:8. doi: 10.1186/s40813-024-00416-3 (PMC11829520; doi:10.1186/s40813-024-00416-3)
Supplement: Supplementary file 1 — Supplementary Material 1: Table 1. Summary of the examined Hungarian farms, sample types and sample sizes. The number and proportion of APPeV-positive samples are also included in the table. [file 40813_2024_416_MOESM1_ESM.pdf]

	Serum				Processing fluid			Oral fluid			Tissue	
Farms	Sample size	Number of pools	Positive sample pools	Proportion of positive sample pools (%)	Sample size	Positive samples	Proportion of positive samples (%)	Sample size	Positive samples	Proportion of positive samples (%)	Yes (Y)/No(N)	Accession number
Farm 1	180	36	6	16,7	10	0	0,0	9	0	0	Y	MH049531-32
Farm 2	100	20	4	20,0	5	5	100,0	10	7	70	Y	MH049527-29
Farm 3	0	0	0	0	0	0	0	0	0	0	N	MH049523-26
Farm 4	0	0	0	0	0	0	0	0	0	0	N	MH049530
Farm 5	80	16	0	0	5	0	0	10	0	0	Y	MH049533
Farm 6	265	53	16	30,2	26	14	53,8	0	0	0	Y	OQ190178
Farm 7	0	0	0	0	21	0	0,0	0	0	0	Y	OQ190180
Farm 8	0	0	0	0	5	3	60	0	0	0	N	N
Farm 9	85	17	0	0,0	3	0	0,0	0	0	0	N	N
Farm 10	70	14	0	0,0	0	0	0	5	1	20	N	N
Farm 11	100	20	6	30,0	8	3	37,5	10	3	30	N	N
Farm 12	60	12	0	0,0	9	0	0,0	4	0	0	N	N
Farm 13	160	32	2	6,3	0	0	0	0	0	0	N	N
Farm 14	130	26	2	7,7	2	0	0,0	10	9	90	N	OQ190183
Farm 15	100	20	0	0,0	5	0	0,0	10	0	0,0	N	N
Farm 16	100	20	4	20,0	5	5	100,0	6	1	16,7	N	OQ190179
Farm 17	100	20	2	10,0	5	0	0,0	10	2	20	N	N
Farm 18	100	20	5	25	5	0	0	10	10	100	Y	OQ190182
Farm 19	100	20	0	0,0	4	0	0,0	9	0	0	N	N
Farm 20	100	20	0	0,0	5	0	0,0	10	0	0	N	N
Farm 21	100	20	4	20,0	5	5	100,0	7	6	85,7	N	OQ190181
Farm 22	100	20	0	0,0	5	1	20,0	9	2	22,2	N	N
Farm 23	70	14	0	0,0	2	0	0,0	10	0	0	N	N
Farm 24	100	20	10	50,0	9	4	44,4	9	5	55,6	N	OQ190176
Farm 25	0	0	0	0	0	0	0	0	0	0	N	Y
Farm 26	0	0	0	0	0	0	0	0	0	0	N	Y
Farm 27	100	20	0	0,0	5	0	0,0	10	2	20	N	N
Farm 28	60	12	0	0,0	5	0	0	10	0	0	N	N
Farm 29	100	20	7	35	5	0	0	10	7	70	N	OQ190177
Farm 30	0	0	0	0	3	0	0	10	4	40	N	N
Farm 31	90	18	0	0	1	0	0	10	1	10	N	N
Farm 32	0	0	0	0	0	0	0	10	0	0	N	N
Farm 33	0	0	0	0	0	0	0	10	0	0	N	N
